# Long COVID: an estrogen-associated autoimmune disease?

**DOI:** 10.1038/s41420-021-00464-6

**Published:** 2021-04-13

**Authors:** Elena Ortona, Danilo Buonsenso, Angelo Carfi, Walter Malorni, The Long Covid Kids study group, Daniel Munblit, Cristina De Rose, Dario Sinatti, Antonia Ricchiuto, Piero Valentini

**Affiliations:** 1 Center for Gender Specific Medicine, Italian National Institute of Health, Rome, Italy; 2 Department of Woman and Child Health and Public Health, Fondazione Policlinico Universitario A. Gemelli IRCCS, Rome, Italy; 3 Dipartimento di Scienze Biotecnologiche di Base, Cliniche Intensivologiche e Perioperatorie, Università Cattolica del Sacro Cuore, Rome, Italy; 4 Center for Global Health, Università Cattolica del Sacro Cuiore, Rome, Italy; 5 Geriatric Department, Fondazione Policlinico Universitario A. Gemelli IRCCS, Rome, Italy; 6 Department of Paediatrics and Paediatric Infectious Diseases, Institute of Child’s Health, Sechcnov First Moscow State Medical University (Sechcnov University), Moscow, Russia; 7 Inflammation, Repair and Development Section, National Heart and Lung Institute, Faculty of Medicine, Imperial College London, London, UK; 8 Research and Clinical Center for Neuropsychiatry, Moscow, Russia

**Keywords:** Diseases, Microbiology

Some people who have had severe to a moderate or mild form of COVID-19 disease may suffer from variable and debilitating symptoms for many months after the initial infection^1^. This condition is commonly called “Long COVID”. An exact definition is missing, but symptoms with a duration of more than 2 months are typically considered as Long COVID. The condition is characterized by long-term sequelae and can involve a range of symptoms such as persistent fatigue, headache, shortness of breath, anosmia, muscle weakness, fever, cognitive dysfunction (brain fog), tachycardia, intestinal disorders, and skin manifestations. Long COVID syndrome bears a similarity to the post-infectious syndromes that followed the outbreaks of chikungunya^2^ and Ebola^3^.

In general, women appear to be twice as likely to develop Long COVID as men, but only until around age 60, when the risk level becomes similar. In addition to being a woman, older age and a higher body mass index also seem to be risk factors for having Long COVID^4^.

## Autoimmune hypothesis and Long COVID

What are the factors responsible for this syndrome? Organ damage caused by an excessive inflammatory response activated by the virus, but also an autoimmune reaction “unmasked” by the virus itself, perhaps due to molecular mimicry with some components of our body, could be responsible for the symptoms of Long COVID^5^. The autoimmune hypothesis could justify the higher incidence of this syndrome in women. In fact, the immune response for both genetic and hormonal factors is stronger in women than in men and this represents a double-edged sword: the outcome of acute COVID-19 is more severe in men but autoimmune reactions are more frequent in women^6,7^. Hence, the study of the appearance of autoantibodies in patient serum and the characterization of the specificity of these autoantibodies could be an important goal to begin to identify personalized and specific treatments also based on the sex of patients affected by Long COVID.

## Long COVID in the child

Recently, the persistence of symptoms following the initial diagnosis of acute COVID-19 has also been demonstrated in the pediatric age^8,9^. In particular, in previous work in a cohort of 129 children with a microbiologically confirmed diagnosis of COVID-19, 27.1% of children have been reported to have at least one symptom more than 120 days after the first diagnosis, whereas three or more symptoms have been reported by 20.6% of children^9^. The most frequent symptoms were muscle and/or joint pain, headache, sleep disturbances, chest pain or chest tightness, palpitations, and sleep disturbances. These symptoms have also been described in children who did not need hospitalization at the time of acute illness or in some with initial asymptomatic SARS-CoV-2 infection. Since sex differences in the occurrence of these symptoms were not considered in the reported study, we now analyzed data disaggregated by sex (Table 1). In general, we found that the majority of symptoms were equally distributed in the two sex, with some exceptions such as headache (16.1 vs. 4.5%), altered smell (6.5 vs. 3%) and taste (4.8 vs 1.5%), and insomnia (22.6 vs. 14.9%), which were more frequently reported in females, although without statistical significance, probably due to the overall low number of children reporting those symptoms. According to our data, a recent preprint assessing the impact of sex in 990 children with acute COVID-19 in Latin America, did not show significant differences^10^

**Table 1:** Details of persisting symptoms of children with COVID-19, according to sex.

Patient and disease features	Total	M	F	*P* value
	*n* = 129	*n* = 67	*n* = 62	
Age	11.0 (4.4)	11.2 (4.5)	10.8 (4.4)	0.585
Symtomatic acute COVID-19	96 (74.4%)	50 (74.6%)	46 (74.2%)	1
Hospitalized during acute COVID-19	6 (4.7%)	5 (7.5%)	1 (1.6%)	0.247
Admitted in PICU	3 (2.3%)	2 (3%)	1 (1.6%)	1
*Timing of survey after acute COVID-19 diagnosis (days)*				0.56
<60	31 (24%)	17 (25.4%)	14 (22.6%)	
60–119	30 (23.3%)	13 (19.4%)	17 (27.4%)	
120 or more	68 (52.7%)	37 (55.2%)	31 (50%)	
*Any persisting symptoms at time of survey*				0.909
None	54 (41.9%)	28 (41.8%)	26 (41.9%)	
1–2	46 (35.7%)	23 (34.3%)	23 (37.1%)	
3 or more	29 (22.5%)	16 (23.9%)	13 (21%)	
*Signs and symptoms reported*				
Fatigue_				0.703
Less	1 (0.8%)	1 (1.5%)	0 (0%)	
A bit less	16 (12.4%)	9 (13.4%)	7 (11.3%)	
Same	98 (76%)	50 (74.6%)	48 (77.4%)	
A bit more	13 (10.1%)	7 (10.4%)	6 (9.7%)	
More	1 (0.8%)	0 (0%)	1 (1.6%)	
Nasal congestion	16 (12.4%)	9 (13.4%)	7 (11.3%)	0.919
Difficulty breathing	8 (6.2%)	4 (6%)	4 (6.5%)	1
Pain on breathing	5 (3.9%)	3 (4.5%)	2 (3.2%)	1
Chest pain	4 (3.1%)	2 (3%)	2 (3.2%)	1
Persistent cough	7 (5.4%)	6 (9%)	1 (1.6%)	0.147
Persistent muscle pain	13 (10.1%)	8 (11.9%)	5 (8.1%)	0.661
Joint pain	9 (7%)	5 (7.5%)	4 (6.5%)	1
Headache	13 (10.1%)	3 (4.5%)	10 (16.1%)	0.057
Altered smell	6 (4.7%)	2 (3%)	4 (6.5%)	0.606
Altered taste	4 (3.1%)	1 (1.5%)	3 (4.8%)	0.557
Lack of concentration	13 (10.1%)	6 (9%)	7 (11.3%)	0.883
Insomnia	24 (18.6%)	10 (14.9%)	14 (22.6%)	0.374
Hypersomnia	4 (3.1%)	3 (4.5%)	1 (1.6%)	0.668
Weight loss	10 (7.8%)	7 (10.4%)	3 (4.8%)	0.389
Poor appetite	10 (7.8%)	6 (9%)	4 (6.5%)	0.84
Diarrhea	2 (1.6%)	1 (1.5%)	1 (1.6%)	1
Abdominal pain	3 (2.3%)	1 (1.5%)	2 (3.2%)	0.946
Constipation	8 (6.2%)	5 (7.5%)	3 (4.8%)	0.801
Skin rash	9 (7%)	6 (9%)	3 (4.8%)	0.568
Palpitations	5 (3.9%)	3 (4.5%)	2 (3.2%)	1
Changes menstruation	2 (1.6%)	0 (0%)	2 (3.2%)	0.442
*Do these symptoms distress your child?*				0.976
Not at all	66 (51.2%)	34 (50.7%)	32 (51.6%)	
Only a little	36 (27.9%)	18 (26.9%)	18 (29%)	
Quite a lot	14 (10.9%)	8 (11.9%)	6 (9.7%)	
A great deal	2 (1.6%)	1 (1.5%)	1 (1.6%)	
Preferred not to say	11 (8.5%)	6 (9%)	5 (8.1%)	
Quality of life Before COVID-19	96.3 (5.3)	96.0 (5.5)	96.7 (5.0)	0.476
Quality of life at the time of the survey	92.9 (9.1)	92.9 (9.1)	92.8 (9.3)	0.987

Although these findings did not show sex differences in pediatric Long COVID, studies on larger cohorts are still needed to achieve stronger conclusions. Conversely, it is also possible that sex differences may be less evident in children compared with adults, due to the lower impact of sex hormones on inflammatory/immune and autoimmune processes in the younger patients. This hypothesis could support the key role of sex hormones in Long COVID clinical features.
